# Impact of primary resistance to front-line targeted therapy in metastatic renal cell carcinoma on subsequent immune-checkpoint-inhibition

**DOI:** 10.1007/s12672-023-00791-3

**Published:** 2023-09-23

**Authors:** J. H. Börner, S. Neuberger, E. Juengel, S. Ziewers, R. Dotzauer, P. Sparwasser, T. Höfner, I. Tsaur, A. Haferkamp, R. Mager

**Affiliations:** Department of Urology and Paediatric Urology, University Medical Center Mainz, Langenbeckstr. 1, 55131 Mainz, Germany

**Keywords:** Metastatic renal cell carcinoma, Resistance, Sequential therapy, Anti-VEGF, Immune checkpoint inhibition

## Abstract

**Introduction:**

Synergistic effects have been discussed for tyrosine kinase (TKI) and immune checkpoint inhibitors (ICI). Primary resistance to TKI might disturb subsequent ICI effectiveness. The objective was to investigate, if primary resistance to 1st line TKI monotherapy predicts response to ICI in subsequent therapy lines and impacts overall survival (OS) in advanced renal cell carcinoma (aRCC).

**Materials and methods:**

Retrospectively, aRCC patients which received front-line TKI from 2016 to 2019 were analyzed for the outcomes primary resistance (1LR), response to sequential ICI therapy, progression free survival (PFS) and overall survival (OS). Kaplan–Meier-estimates, Cox proportional hazards and logistic regression were used.

**Results:**

Primary resistance to front-line TKI was observed in 27 (53%) of 51 patients. Groups with disease control (DC) and 1st line TKI resistance (1LR) were not different at baseline with regard to clinicopathological features. Median duration on 1st line therapy was significantly shorter in the 1LR (5.1 months) than in the DC (14.7 months) group (p = 0.01). Sequential therapy was started in 21 (75%) and 12 (52%) patients of 1LR and DC groups using nivolumab in 16 (76%) vs. 11 (92%) cases (p > 0.05). Logistic regression revealed that 1LR status, neutrophil-to-lymphocyte ratio < 3, IMDC favorable prognosis and clear cell histology had no significant impact on responsiveness to ICI in subsequent therapy lines. Cox proportional hazards demonstrated no significant association of 1LR status with PFS and OS in patients who received subsequent ICI treatment.

**Conclusion:**

Primary TKI resistance of aRCC was neither significantly associated with responsiveness to ICI during sequential therapy nor with PFS and OS. This adds the evidence for ICI based sequential therapy in primary TKI resistant aRCC.

**Supplementary Information:**

The online version contains supplementary material available at 10.1007/s12672-023-00791-3.

## Introduction

In 1st line therapy of advanced renal cell carcinoma combination therapy using tyrosine kinase inhibitors (TI) and immune checkpoint inhibition (ICI) proved to be superior to TKI monotherapy with sunitinib in 3 pivotal phase III studies [1–3]. Extended follow-up data including subsequent therapy lines demonstrated significant overall survival (OS) advantage for patients with upfront combination regimes [2, 4]. Favorable synergistic effects known from various types of solid cancer have been discussed to explain the superiority of combination regimes over TKI monotherapy [5–8]. However, in the same way, tumors harboring primary resistance against anti-VEGF TKI lack from these effects and that might result in reduced activity of the combination agents or of subsequent agents. Although molecular characterization of advanced renal cell carcinoma (aRCC) has led to an increased understanding of the disease targets, the individual efficacy of currently available drugs remains unpredictable [9]. Primary resistance against TKI occurring in approximately 30% of patients is known to result in poor prognosis due to limited response to subsequent therapy with VEGF-targeted TKIs or mammalian target of rapamycin (mTOR) inhibitors [10–12]. Naturally, these patients are still present in the era of 1st line combination therapy with ICI and TKI, but the lack of responsiveness to TKI might be superimposed by the efficacy of the immune checkpoint inhibitor. However, the impact of primary resistance to upfront TKI on oncological outcome parameters in the era of ICI is unclear and the need for data has already been addressed [6, 13, 14]. The analysis of cases harboring primary resistance to anti-VEGF drugs in the setting of subsequent ICI exposition might provide further insights in the mechanisms of simultaneous efficacy of the current 1st line combination therapy standard. Furthermore, there is still uncertainty about 1st line combination regimes in the elderly with regard to toxicity and less pronounced survival benefit compared to TKI monotherapy [15]. Thus, the primary endpoint of this work was to assess, if primary resistance to first-line TKI monotherapy predicts response to ICI in subsequent therapy lines. The secondary outcome measures included the examination, if primary resistance to first-line TKI monotherapy influences progression free survival and overall survival.

## Methods

### Design and patients’ selection

A retrospective analysis of a prospectively collected aRCC database of the universitary cancer center Mainz was performed. Cases were recorded beginning 2016 to cover the era of ICI treatment in aRCC. Inclusion of the last case was 2020, when 1st line therapy increasingly changed to combination regimes. Data acquisition and analysis adhered to local ethical standards [REC 83755015 (1018) and 2022-16461]. Inclusion criteria were histologically proven RCC, radiologically proven metastatic state, age ≥ 18 years and to have received at least 4 weeks of 1st line (1L) TKI treatment.

### Treatment and follow-up

Patients received TKI and ICI treatment in an outpatient setting and got clinical check-up every four weeks. If toxicity of 1st line TKI drug occurred within the first 2 months, changing to another TKI approved for 1st line treatment was allowed without new restaging. In general, according to the local standard of care, restaging was performed every three months after initiation of a new therapy line. Imaging reporting followed RECIST 1.1 criteria [16]. Patients fulfilling disease control (DC) criteria (complete remission, partial remission or stable disease) at first restaging 3 months after initiation of treatment were assigned to “1DC” group. Patients with immediate disease progression recorded at first restaging after the first 3 months of therapy were defined as primary resistant to first-line, referred to as “1LR”. The multidisciplinary genitourinary cancer board was consulted standardly at initiation of treatment or at every switch to the next therapy line, e.g. at disease progression or in case of intolerable toxicity. In some patients of the 1LR cohort, the multidisciplinary genitourinary cancer board decided to wait for the 2nd restaging at 6 months to avoid the misinterpretation of cases with pseudo-progression. Outside of active treatment, i.e. in cases changing to best supportive care, follow-up data were requested quarterly at the primary care physician.

### Statistical analysis

Comparison of the two groups included clinicopathologic features, primary resistance to 1st line substance and disease control by ICI during sequential therapy. Categorical variables were analyzed by Chi-Square or Fisher’s test. Continuous variables were assessed using Mann–Whitney-U test. Kaplan–Meier estimates and Cox proportional hazards were used to examine duration on 1st line therapy, PFS and OS. Cases, where death did not occur during the time observed, were censored.

Based on their evidence to be associated with oncological outcomes of aRCC, the variables clear cell histology, International Metastatic Renal-Cell Carcinoma Database Consortium (IMDC) favorable prognosis group and neutrophil-to-lymphocyte ratio (NLR) < 3 were compared with 1DC status to elucidate predictors for efficacy of ICI, i.e. addressing the primary outcome measure, by logistic regression analysis [17–19]. An additional exploratory analysis examined those cases, which had never received ICI treatment during sequential therapy. The level of significance was set to p < 0.05. Bias software (epsilon, Frankfurt, Germany) was used for statistical analysis [20].

## Results

In total 51 patients received 1st line treatment of aRCC. Disease control was recorded in 24 (47%) patients. The 1DC (1st line disease control) group did not differ significantly from those 27 (53%) patients, who had immediate progression of disease, in terms of clinicopathologic features at baseline (Table 1).

**Table 1:** Patients’ baseline characteristics

	1DC group N = 24	1LR group N = 27	p value
Age, y^a^	72 [60–79]	67 [58–74]	0.4
Male/female, n/n (%/%)	19/5 (79/21)	21/6 (78/22)	1.0
IMDC prognosis group			
Favorable, n (%)	5 (21)	5 (18)	
Intermediate, n (%)	18 (75)	15 (56)	
Poor, n (%)	1 (4)	7 (26)	0.08
Synchronous M+, n (%)	7 (29)	8 (30)	1.0
s/p nephrectomy, n (%)	24 (100)	26 (96)	1.0
Clear cell histology, n (%)	22 (92)	21 (78)	0.3
T1, n (%)	3 (13)	6 (22)	0.6
T2, n (%)	6 (25)	5 (18)	0.8
T3, n (%)	15 (62)	15 (56)	0.8
T4, n (%)	0	1 (4)	1.0
CCI ≥ 9, n (%)	11 (46)	13 (48)	1.0
Organ systems affected by metastases^a^	2 [1, 2]	2 [1–3]	0.3
Pulmonary, n (%)	18 (75)	17 (63)	0.5
Bone, n (%)	6 (25)	10 (37)	0.5
Lymph node, n (%)	6 (25)	10 (37)	0.5
Soft tissue, n (%)	3 (13)	6 (22)	0.5
Skin, n (%)	0	1 (4)	1.0
Cerebral, n (%)	0	1 (4)	1.0
Pancreatic, n (%)	1 (4)	1 (4)	1.0
Hepatic, n (%)	2 (8)	7 (26)	0.1
Adrenal, n (%)	2 (8)	0	0.2
Serosa, n (%)	0	3 (11)	0.2
Neutrophil to lymphocyte ratio^a^	3.2 [2.7–4.8]	3.2 [2.6–6.0]	0.9

*DC* disease control, *1LR* 1st line resistant, *n* number, *IMDC* International Metastatic Renal-Cell Carcinoma Database Consortium, *s/p* status post, *CCI* Charlson Comorbidity Index, *M*+ distant metastases

^a^Variables presented as median, interquartile range in brackets

A detailed breakdown of first line and sequential therapies is presented in Table 2.

**Table 2:** Sequential therapy

	1DC group N = 24	1LR group N = 27	p value
First line (1L) therapy, n (%)			
Sunitinib, n (%)	11 (46)	18 (66)	0.2
Pazopanib, n (%)	6 (25)	7 (26)	1.0
Cabozantinib, n (%)	6 (25)	1 (4)	0.04
Tivozanib, n (%)	1 (4)	1 (4)	1.0
Duration on 1L, months	14.7	5.1	0.01
Dose intensity, d_100%_ (%)	10,168 (54)	10,965 (60)	0.05
CTCAE any grade during 1L, n (%)	11 (46)	9 (33)	0.5
CTCAE grade ≥ 3 during 1L, n (%)	3 (13)	6 (22)	0.5
Start of sequential therapy, n (%)	12 (50)	21 (78)	0.07
Drugs used for sequential therapy, n/n (%)			
Nivolumab (ICI)	11/12 (92)	16/21 (76)	0.4
Cabozantinib (TKI)	3/12 (25)	7/21 (33)	0.7
Lenvatinib + everolimus (TKI + mTor-I)	0 (0)	6/21 (29)	0.06
Axitinib (TKI)	2/12 (17)	3/21 (14)	1.0
Tivozanib (TKI)	0 (0)	2/21 (10)	0.5
Sorafenib (TKI)	0 (0)	1/21 (5)	1.0
Sunitinib (TKI)	5/12 (42)	0 (0)	0.01
Pazopanib (TKI)	3/12 (25)	0 (0)	0.04
Everolimus (mTor-I)	2/12 (17)	0 (0)	0.1
Temsirolimus (mTor-I)	0 (0)	1/21 (5)	1
Number of therapeutic sequences after 1L^a^	0.5 [0–2]	1 [0–2]	0.3

*CTCAE* common criteria of adverse events, *TKI* tyrosine kinase inhibitor, +*mTor-I* mammalian target of rapamycin inhibitor, *d*_*100%*_ days on treatment with 100% dose

^a^Variables presented as median, interquartile range in brackets

Cabozantinib was significantly more often administered in 1DC compared to 1LR group (p = 0.04). Naturally, efficacy of 1st line TKI resulted in significantly longer duration on 1st line therapy, compared with 1LR patients who suffered from immediate progression of disease. With regard to toxicity of 1st line TKI, there was no difference of grade ≥ 3 adverse events between 1DC and 1LR groups. At a median follow-up of 39 months, 50% and 78% of 1DC and 1LR groups have started with subsequent therapy lines. Off these, 27 patients received Nivolumab whereas 24 patients never got ICI treatment during the time observed. Combination treatment with lenvatinib and everolimus during subsequent therapy has been administered exclusively to patients with primary resistance. Notably, one chromophobe aRCC patient with primary TKI and primary ICI refractory disease achieved disease control with lenvatinib and everolimus.

The baseline and treatment characteristics of those, who received ICI treatment during sequential therapy, are depicted in Table 3. Disease control did not differ significantly between groups. Immune related adverse events ≥ grade 3 occurred significantly more often among 1DC compared to 1LR patients.

**Table 3:** Baseline and treatment characteristics of patients, who received ICI treatment during sequential therapy

	1DC group N = 11	1LR group N = 16	p value
Baseline characteristics			
Age, y^a^	73 [64–76]	65 [60–73]	0.2
Male/female, n/n (%/%)	9/2 (82/18)	14/2 (88/12)	1.0
IMDC prognosis group			
Favorable, n (%)	2 (18)	3 (19)	1.0
Intermediate, n (%)	9 (82)	11 (69)	0.8
Poor, n (%)	0	2 (12)	0.6
Synchronous M+, n (%)	2 (18)	5 (31)	0.8
s/p nephrectomy, n (%)	11 (100)	15 (94)	1.0
Clear cell histology, n (%)	10 (91)	13 (81)	0.6
T1, n (%)	2 (18)	4 (25)	0.6
T2, n (%)	2 (18)	3 (19)	0.8
T3, n (%)	7 (64)	9 (56)	0.8
T4, n (%)	0	0	1.0
CCI ≥ 9, n (%)	5 (45)	7 (44)	1.0
Neutrophil to lymphocyte ratio^a^	3.6 [2.8–3.1]	3.0 [2.1–4.7]	0.2
Disease control and toxicity of sequential therapy			
Disease control by ICI	4 (36)	7 (44)	0.7
irAE any grade during ICI	6 (55)	5 (31)	0.3
irAE Grade ≥ 3 during ICI	5 (45)	1 (6)	0.02
Disease control by TKI after ICI	2 (18)	4 (25)	1.0

*DC* disease control, *1LR* 1st line resistant, *n* number, *IMDC* International Metastatic Renal-Cell Carcinoma Database Consortium, *s/p* status post, *CCI* Charlson Comorbidity Index, *M*+ distant metastases, *irAE* immune related adverse events, *ICI* immune checkpoint inhibition, *TKI* tyrosine kinase inhibitor

^a^Variables presented as median, interquartile range in brackets

A detailed breakdown of adverse events is presented in Additional file 1.

Progression free survival after initiation of ICI treatment during sequential therapy did not differ between the 1DC group, with a median PFS of 5.2 months (95%-KI = [2.1; 8.3]), and the 1LR group, with a median PFS of 4.5 months (95%-KI = [3.0; 6.0]) and Cox proportional hazards demonstrated no significant association of 1st line resistance to TKI with PFS in these patients (Relative hazards 1.2, 95%-confidence interval 0.5; 2.8, p = 0.7).

**Fig. 1:**
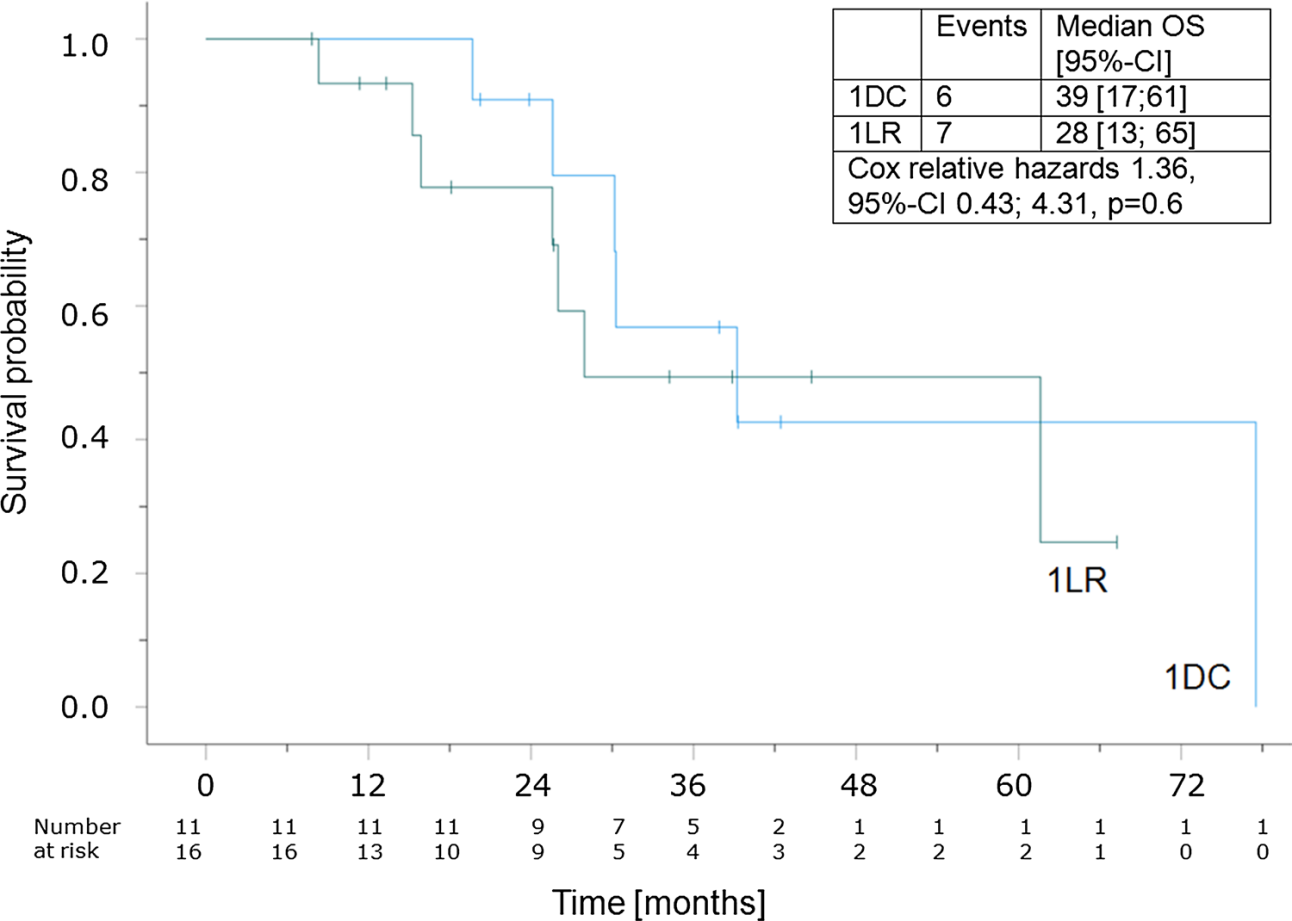
The Kaplan–Meier-Curve demonstrates the survival probability of the 1DC (1DC = 1st line disease control group) vs. the 1LR group (1LR = primary resistance to 1st line TKI treatment). The observation time begins at initiation of 1st line TKI treatment. Median follow-up was 39 months. The median overall survival of the 1DC group was 39 months (95%-confidence interval 17; 61) (6 events) and of the 1LR group 28 months (95%-CI 13; 65) (7 events). Censored data is depicted as short vertical line

Kaplan–Meier estimates for OS of those, who received ICI treatment during sequential therapy, are depicted in Fig. 1. Cox proportional hazards revealed no significant association of 1st line resistance to TKI with OS in patients, that received subsequent ICI treatment (Relative hazards 1.36, 95%-confidence interval 0.43; 4.31, p = 0.6), but demonstrated a significant association with poorer OS in those, who never received ICI treatment (Relative hazards 6.56, 95%-confidence interval 1.67; 25.67, p < 0.007). Univariable und multivariable Logit model including clear cell histology, IMDC favorable prognosis group, NLR < 3 and 1DC status failed to reveal an independent predictor for clinical efficacy of ICI (Table 4).

**Table 4:** Univariable and multivariable logistic regression analysis for overall responsiveness to ICI (nivolumab) during sequential therapy

Variable	Odds ratio [95%-CI]	p value
Univariable logistic regession		
NLR < 3	1.1 [0.2; 5.8]	0.9
IMDC favorable prognosis	2.6 [0.3; 20.7]	0.4
ccRCC	2.3 [0.2; 28.2]	0.5
1st line primary resistance	1.4 [0.3; 7.0]	0.7
Multivariable logistic regression		
NLR < 3	1.3 [0.2; 8.5]	0.8
IMDC favorable prognosis	4.8 [0.3; 78.4]	0.3
ccRCC	2.6 [0.2; 42.0]	0.5
1st line primary resistance	2.1 [0.3; 15.1]	0.4

*NLR* neutrophil to lymphocyte ratio, *ccRCC* clear cell renal cell carcinoma

## Discussion

The current work provides real world data of aRCC patients with primary resistance to 1st line TKI and its impact on responsiveness to ICI in subsequent therapy lines compared to patients achieving disease control under 1st line therapy. In real world data primary resistance to TKI has been reported in 26–29% of cases which is much lower than demonstrated in our patient population [11, 12]. Since the respective baseline characteristics appear to be roughly equal a more lenient clinical judgment of the Karnovsky performance score (KPS) by our attending physicians might be a possible explanation. Furthermore, an impaired KPS and—except from hypercalcemia—all the other IMDC risk factors have been demonstrated to be independently associated with primary refractory disease. Nevertheless, although a numeric larger number of poor prognosis group patients were recorded in our 1LR group, this difference to 1DC group was not significant [11].

Data addressing the issue of primary resistance in the era of ICI is even sparser. Before ICI was introduced, primary TKI refractory disease resulted in poor median OS ranging from 6.8 to 14.9 months [10, 11]. Nowadays, after ICI and powerful multi-kinase inhibitors such as cabozantinib and lenvatinib have been established for sequential therapy, our cohort of primary anti-VEGF-therapy resistant patients demonstrates a considerably improved median OS of 28 months. Interestingly, these 16 patients exceed the overall survival reported by Hamie et al. in 4 patients with primary resistance to TKI ranging from 4 to 17 months in the same therapeutic era [13].

Pro-immunogenic effects by hindering tumor escape mechanisms have been suggested for TKI [5, 7]. Our data demonstrated that the lack of these mechanisms to target the tumor in primary TKI resistant RCC was not significantly associated with the susceptibility to the subsequently targeted PD-1 immune checkpoint mechanisms. Interestingly, exposure to ICI might have supported overcoming both primary and acquired resistance in a couple of patients. This finding also supports the current 1st line standard to combine TKI and ICI. The underlying mechanisms for these effects are still unclear, but targeting of AXL which promotes tumor immune evasion and therefore has been discussed to play a role in PD-1 resistance, might be one possible explanation [21]. Another potential target that could be linked with PD-1 resistance might include KIT as an important player in the tumor microenvironment [13, 22]. Furthermore, to target also the fibroblast growth factor receptor (FGFR) with lenvatinib has demonstrated additional anticancer activity by restoring the tumor response to IFNγ stimulation in a current preclinical study [23].

NLR as an easily measurable biomarker demonstrated to predict outcome in various cancers. Although a higher NLR was associated with poorer outcomes, there is no validated cut-off value distinguishing high from low, and therefore varies in literature [17, 24–26]. Recently Pham et al. demonstrated a poorer OS for NLR ≥ 3 in 36 aRCC patients receiving ICI. Following this cut-off value, in our cohort NLR < 3 demonstrated no significant association with response to ICI. Interestingly, although the IMDC risk score is an established predictor for OS in aRCC, there was no significant association with response to ICI in our population. No new toxicity signs were demonstrated by our data and the frequency of adverse events was in the expected range [27].

The work is limited by its retrospective nature and its relatively small sample size. However, to date, the current work represents one of the largest series of real world data analyzing primary TKI resistant aRCC cases in comparison to primary TKI responders with regard to responsiveness to sequential ICI therapy.

## Conclusion

With the current real world data we strengthen the evidence for sequential therapy in patients with aRCC harboring primary resistance to 1st line TKI. Although duration on 1st line therapy was significantly shorter and overall survival was numerically inferior in aRCC patients with primary TKI resistance, overall survival and responsiveness to ICI in subsequent therapy lines were not significantly associated with 1st line TKI resistance. Obviously, despite the lack of antiangiogenic mediated immunomodulatory mechanisms of the failed 1st line approach, ICI can overcome resistance by approaching different targets.

Therefore, in primary resistant aRCC, sequential therapy including ICI should be started whenever possible, since there is an individual and unpredictable new chance for disease control and for catching up with the primary responders’ OS outcomes.

### Supplementary Information

**Additional file 1.** Adverse events of 1st line TKI therapy and of ICI during sequential therapy.

## Data Availability

Data available on request.
