# Supplementary material for: Impact of primary resistance to front-line targeted therapy in metastatic renal cell carcinoma on subsequent immune-checkpoint-inhibition
Source: Discov Oncol. 2023 Sep 23;14:178. doi: 10.1007/s12672-023-00791-3 (PMC10517909; doi:10.1007/s12672-023-00791-3)
Supplement: Supplementary file 1 — Additional file 1. Adverse events of 1st line TKI therapy and of ICI during sequential therapy. [file 12672_2023_791_MOESM1_ESM.docx]

**Additional material.** Adverse events of 1^st^ line TKI therapy and of ICI during sequential therapy.

| Adverse events of 1^st^ line TKI therapy | | |
| --- | --- | --- |
|  | 1DC group n = 24 | 1LR group n = 27 |
| Pneumonia | 1 |  |
| Pleural effusion |  | 1 |
| Pneumonitis | 1 |  |
| Enteritis |  | 1 |
| Diarrhea | 2 |  |
| Acute heart failure |  | 1 |
| Fatigue | 3 | 2 |
| Edema |  | 1 |
| Stomatitis | 1 |  |
| Cutaneous | 1 | 2 |
| Hyponatremia |  |  |
| Hoarseness | 1 | 1 |
| Vasculitis | 1 |  |
|  |  |  |
| Immune related adverse events of ICI during sequential therapy | | |
|  | 1DC group n = 11 | 1LR group n = 16 |
| Pneumonitis |  | 1 |
| Pulmonary abszess | 1 |  |
| Acute renal failure |  | 1 |
| Myalgia | 1 |  |
| Diarrhea | 2 | 1 |
| Pancreatitis | 1 |  |
| Dermatitis |  | 1 |
| Fatigue | 1 |  |
| Hypothyreosis |  | 1 |
|  |  |  |
